# Exploring global perspectives on the use of computer-based simulation in pharmacy education: a survey of students and educators

**DOI:** 10.3389/fphar.2024.1494569

**Published:** 2024-10-15

**Authors:** Ahmed M. Gharib, Ivan K. Bindoff, Gregory M. Peterson, Mohammed S. Salahudeen

**Affiliations:** School of Pharmacy and Pharmacology, College of Health and Medicine, University of Tasmania, Hobart, TAS, Australia

**Keywords:** computer-based simulation, pharmacy education, educational technology, curriculum integration, simulation-based learning, virtual patients

## Abstract

**Background:**

: Increasing student numbers and logistical challenges in pharmacy education limit patient counselling and clinical placement opportunities. Computer-based simulation (CBS) offers scalable, interactive learning but faces integration barriers.

**Objective:**

: To explore global perceptions of CBS implementation in pharmacy education among educators and students. Methods: An online cross-sectional survey was developed based on literature review and expert feedback. The survey was distributed globally through academic pharmacy organisations, social media, and the authors’ networks. It included 20 questions targeting pharmacy educators and students.

**Results:**

: Responses from 152 educators across 38 countries and 392 students from 46 countries, spanning six WHO regions (AFRO, AMRO, EMRO, EURO, SEARO, and WPRO) were analysed using descriptive and inferential statistics. The majority of educators (90.1%, n = 137) and students (84.2%, n = 330) expressed comfort with using CBS and implementing it in their curriculum. Despite this, CBS was perceived as underutilised by 53.5% (n = 81) of educators and 63.7% (n = 250) of students. Students valued CBS for enhancing communication and problem-solving skills, while educators highlighted its relevance to community pharmacy practice. Both groups supported CBS use in assessments. All educators (100%) identified workload reduction as a key priority, hoped CBS could assist in this area. Educators also reported barriers such as financial constraints (56.6%, n = 86) and insufficient technical support (53.3%, n = 81). On the other hand, students were less optimistic about institutional support, with only a few (7.4%, n = 29) believed institutional leaders would actively support CBS adoption. Regional differences emerged, with SEARO (Southeast Asia) and AFRO (Africa) showing the lowest CBS usage rates. Educators in SEARO, AFRO, and EMRO (Eastern Mediterranean) raised concerns about technical support, while those in SEARO, AFRO, and WPRO (Western Pacific, including Australia, New Zealand, and Singapore) expressed financial concerns. Educators in AFRO and WPRO, however, reported being 100% comfortable with using CBS.

**Conclusion:**

: Both students and educators recognised the potential of CBS in pharmacy education, with strong support for its integration. Addressing barriers such as educator workload, financial constraints, and technical support is crucial for broader adoption. Improved resource allocation and targeted training for educators are essential to effectively incorporate CBS into the pharmacy curriculum.

## 1 Introduction

In pharmacy education, experiential learning through standardised patients, Observed Structured Clinical Examinations (OSCEs), and classroom-based simulations has been essential for developing practical skills and bridging theoretical knowledge with real-world application (Gharib et al., 2023a). However, the increasing number of pharmacy students and associated logistical and financial constraints have strained traditional placement opportunities, highlighting the need for alternative, scalable methods to complement experiential learning (Hall et al., 2012; Mai et al., 2019).

Computer-based simulation (CBS) represents a promising solution in healthcare education, offering virtual simulations of clinical scenarios that allow users to replicate the roles and responsibilities of healthcare professionals in a controlled, risk-free environment (Mai et al., 2019; Cook et al., 2011; Benedict and Schonder, 2011; Duff et al., 2016; Seybert et al., 2019). This technology not only provides a scalable and interactive platform for repeated practice and immediate feedback to students (Gharib et al., 2023a; Jabbur-Lopes et al., 2012), but also aligns with Kolb’s experiential learning theory by fostering critical thinking and clinical reasoning through active problem-solving and decision-making (Ried et al., 2002; Looyestyn et al., 2017; Jaber and Al-worafi, 2023). Additionally, CBS enhances communication and teamwork skills by simulating interdisciplinary interactions and patient counselling (Rash et al., 2024; Phanudulkitti et al., 2023).

Despite the availability of various CBS solutions in the market, their utilisation in pharmacy education remains relatively low. There is a significant gap in the literature concerning the perspectives of key educational stakeholders, including educational institutions, educators, and students, on the barriers and facilitators to CBS implementation in pharmacy education. Distinct stakeholders may have varying needs, which could directly or indirectly influence the success of the implementation process (DiVall et al., 2014). While the benefits of CBS in healthcare education are well-documented, comprehensive global data on its use in pharmacy education remain scarce. Most of the existing research focuses on individual institutions or specific tools, lacking a broader perspective on the challenge and enablers of CBS adoption across different global regions.

This study aims to fill the gap by exploring the global views of both students and educators on CBS usage across six WHO regions. The insights gained are expected to provide valuable guidance to stakeholders in pharmacy education, potentially influencing future practices and policies related to CBS. By addressing this research gap, the study seeks to contribute to the advancement of pharmacy education through the adoption of innovative and effective teaching methods.

## 2 Methods

**FIGURE 1:**
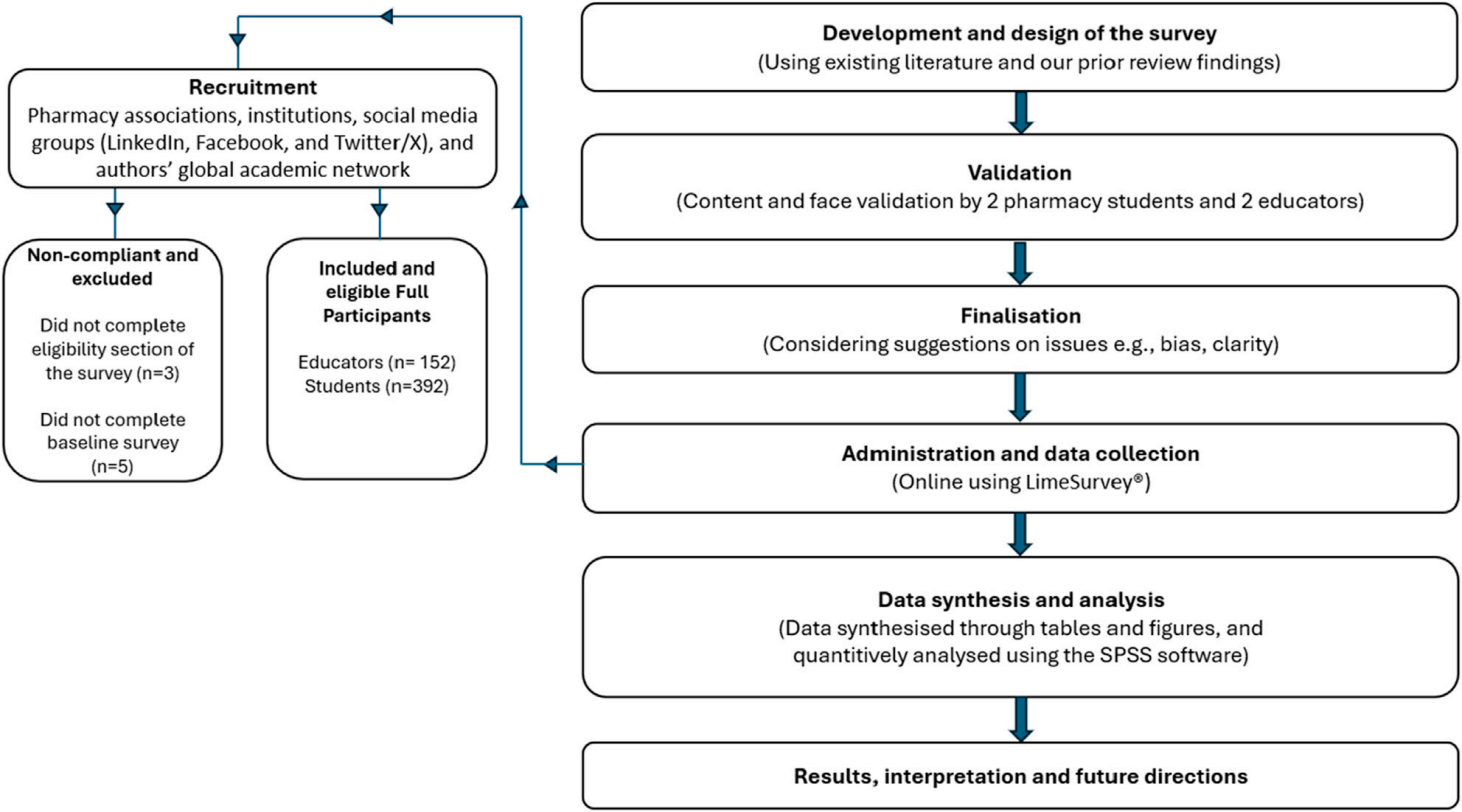
Survey process flowchart.

Between March and September 2023, two parallel, cross-sectional, mixed-methods online surveys were conducted to explore global perspectives on CBS in pharmacy education. One survey targeted pharmacy practice educator, and the other targeted students. The surveys followed an exploratory approach to align with the study’s objectives (Watling and Lingard, 2012; Jansen, 2010; Guetterman et al., 2019), as described in Figure 1.

### 2.1 Development of the survey

Survey questions were developed based on a comprehensive literature review (Gharib et al., 2023a; Gharib et al., 2023b) and iterative scoping team meetings. The final version of each 20-item survey across four sections was designed to assess views on using CBS technology, perceived implementation barriers, and general interest in integrating CBS within the curriculum. The surveys are detailed in Supplementary Appendix SA, SB.

Section 1 of the survey focused on general information, demographics, and background on CBS usage if applicable. Additional questions were asked for participants who indicated previous experience using CBS. Section 2 involved rating the importance of various elements related to CBS. Section 3 examined CBS experience, if applicable, with an additional option for participants wishing to provide more details. Section 4 covered perceived support and barriers to CBS implementation, with additional options for participants who wanted to provide more details. The draft survey underwent content validation by two pharmacy students and two pharmacy educators. To ensure confidentiality and anonymity, no personal details of the participants were collected. Surveys were administered in English, using LimeSurvey^®^ (LimeSurvey GmbH, Hamburg, Germany. URL http://www.limesurvey.org).

### 2.2 Recruitment

Eligible participants included pharmacy practice educators involved in teaching relevant courses and pharmacy students enrolled in pre-registration degree programs. No restrictions were placed on prior CBS experience or language of course instruction, provided participants could complete the survey in English.

The surveys aimed to capture responses from across the six World Health Organisation (WHO) regions: African Region (AFRO), Region of the Americas (AMRO), Eastern Mediterranean Region (EMRO), European Region (EURO), South-East Asia Region (SEARO), and Western Pacific Region (WPRO) (WorldHealthOrganization, 2024). The survey links were distributed through different pharmacy student associations and social media groups for pharmacy students and educators (LinkedIn, Facebook, and Twitter/X), as well as the authors’ global academic network. An information sheet on the survey cover page provided general information about the study, including eligibility. Participation was voluntary, and completion of the survey was deemed as implied consent. All participants who completed the survey were entered in a draw to win one of ten $50 AUD gift vouchers per survey. All completed questionnaires were reviewed for eligibility and completeness.

### 2.3 Analysis

Data were analysed using SPSS (IBM Corp. Released 2012. IBM SPSS Statistics for Windows, version 26.0. Armonk, NY, United States: IBM Corp.) and Microsoft Office Excel. Descriptive statistics were employed to summarise the quantitative data. Inferential statistics, such as chi-square tests for categorical variables, were used to compare responses between the educators and students. A *p*-value of < 0.05 was considered statistically significant for all analyses.

Ethical approval was obtained from the University of Tasmania’s (UTAS) Human Research Ethics Committee (Project ID: 26897).

## 3 Results

**FIGURE 2:**
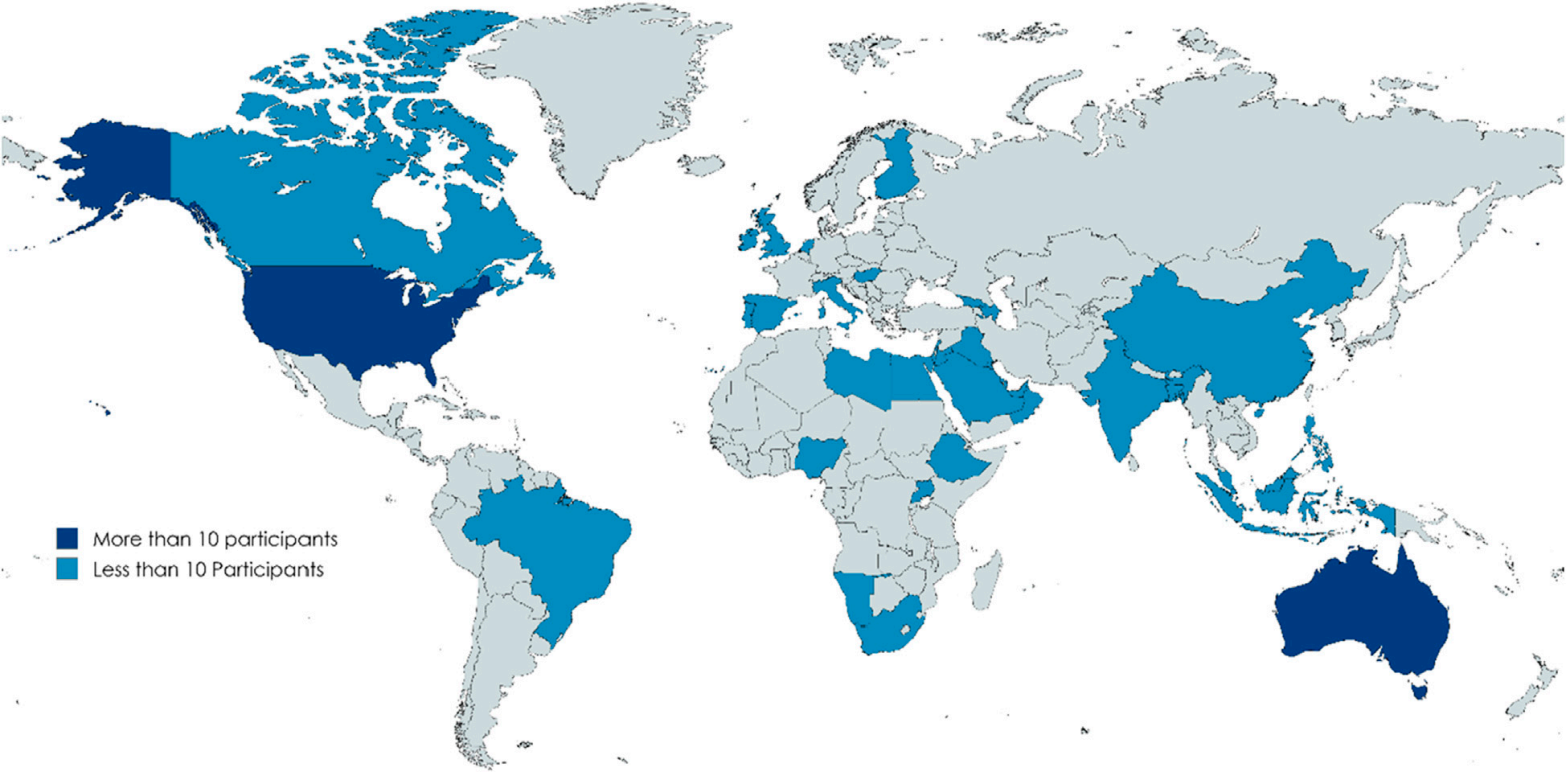
Distribution of the study participants (Educators) Region of the Americas (AMRO): United States of America, Canada, Brazil. South-East Asia Region (SEARO): India, Indonesia, Bangladesh. European Region (EURO): Ireland, United Kingdom, Portugal, Spain, Finland, Italy, Hungary, Netherland, Georgia, Azerbaijan. Eastern Mediterranean Region (EMRO): Israel, Jordan, Bahrain, Qatar, Kuwait, Oman, Libya, Kingdom of Saudi Arabia, Iraq, Lebanon, United Arab Emirates, Egypt. African Region (AFRO): Ethiopia, Nigeria, South Africa, Uganda, Namibia. Western Pacific Region (WPRO): Australia, Malaysia, Philippines, Singapore, China.

**FIGURE 3:**
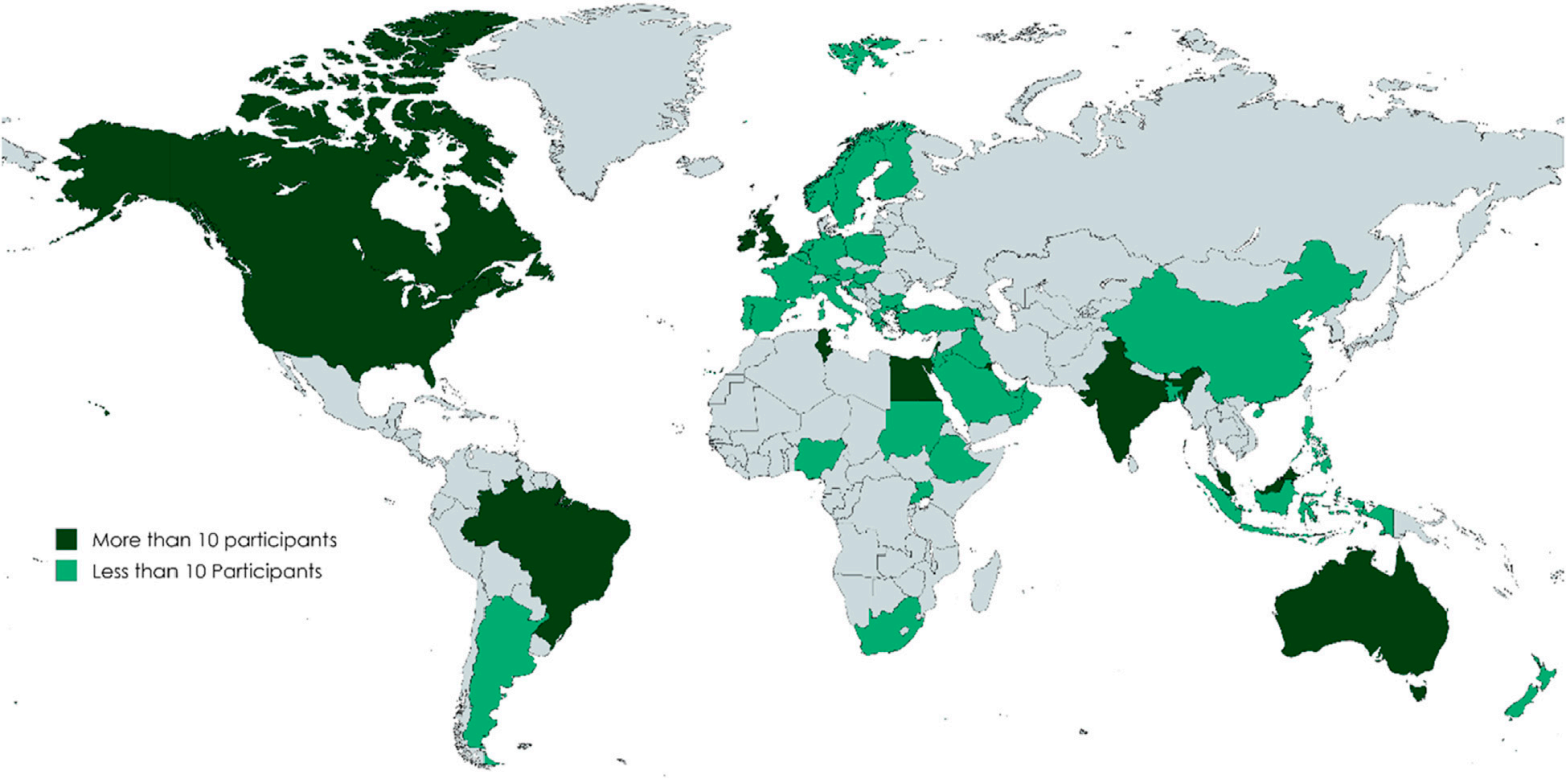
Distribution of the study participants (Students) Region of the Americas (AMRO): United States of America, Canada, Brazil, Argentina. European Region (EURO): Ireland, United Kingdom, Belgium, France, Germany, Sweden, Netherlands, Greece, Austria, Bulgaria, Poland, Portugal, Italy, Norway, Croatia, Turkey, Armenia. South-East Asia Region (SEARO): India, Indonesia, Bangladesh. Eastern Mediterranean Region (EMRO): Egypt, Lebanon, Kuwait, Kingdom of Saudi Arabia, Qatar, Jordan, United Arab Emirates, Oman, Iraq, Bahrain, Tunisia, Sudan. African Region (AFRO): Ethiopia, Uganda, Nigeria. South Africa. Western Pacific Region (WPRO): Australia, Malaysia, Singapore, New Zealand, Philippines, China.

The surveys captured a comprehensive global perspective, gathering insights from 152 educators across 38 countries and 392 students from 46 countries (see Figures 2, 3). The largest group of educator participants was from (AMRO) with (33.6%, n = 51), while (AFRO) had the lowest representation of (5.9%, n = 9). Among students, EMRO had the highest participation of 100 students (25.5%), while the AFRO again underrepresented with 21 students (5.4%), see Table 1.

**TABLE 1:** Regional distribution of surveys’ participants (Students and Educators).

Metric / Region	Region of the Americas (AMRO)	South-East Asia Region (SEARO)	European Region (EURO)	Eastern Mediterranean Region (EMRO)	African Region (AFRO)	Western Pacific Region (WPRO)
Educators Survey Participants Total =152 participants n (%)	51 (33.6%)	15 (9.9%)	25 (16.4%)	30 (19.7%)	9 (5.9%)	22 (14.5%)
Students Survey Participants Total =392 participants n (%)	74 (18.9%)	30 (7.7%)	90 (23%)	100 (25.5%)	21 (5.4%)	77 (19.6%)

The educators’ teaching experience was fairly balanced, with (51.3%, n = 78) having more than 5 years of teaching experience, and (48.7%, n = 74) having 5 years or less. Students tended to be more experienced, with (73.5%, n = 288) having less than 2 years remaining in their pharmacy program, and only (26.5%, n = 104) having more than 2 years left.

### 3.1 General perceptions across all participants

Both educators and students exhibited high levels of comfort with using CBS. Among educators, (90.1%, n = 137) strongly agreed/agreed with the statement, *“I would be comfortable using computer-based technology in teaching”*, while (84.2%, n = 330) similarly strongly agreed/agreed with their equivalent statement. Disagreement was minimal, reported by (7.2%, n = 11) educators and (2.6%, n = 10) students.

Regarding the desire to use CBS for assessing knowledge, students showed a higher level of interest compared to educators. Specifically, (71.9%, n = 282) students strongly agreed/agreed with the statement, *“I would like to use a computer-based simulation tool to assess my knowledge”,* while (56.6%, n = 86) educators expressed a similar interest in using CBS to assess their students’ knowledge.

Student interest in using CBS within the classroom setting was mixed. For the statement, *“I would like to use a computer-based simulation tool in the classroom”,* (52.6%, n = 206) students strongly agreed/agreed, indicating a moderate level of interest in integrating CBS directly into classroom activities. In contrast, a stronger preference was observed for using CBS as a supplementary tool, with (77.8%, n = 305) students strongly agreeing/agreeing with the statement, *“I would like to use a computer-based simulation tool to supplement my classroom study”.*

When participants were asked to rate the importance of various elements in CBS training, skills such as patient communication, counselling, and problem-solving were widely recognised as very important by both educators and students (see Table 2). Interestingly, both groups placed significant emphasis on community pharmacy and hospital pharmacy as the preferred settings for delivering CBS skills. However, opinions on the importance of community clinic practice training were more varied, with (23.7%, n = 36) educators considering it “Not Important at All” compared to (9.4%, n = 37) students (see Table 2).

**TABLE 2:** Participants (Students and Educators) perspectives on CBS training preferences (in response to the question: indicate how you rate the importance of each of the following elements).

CBS training focus Statements “When considering the training focus for a CBS: indicate how you rate the importance of each of the following elements?”	Students’ perspectives (Total number = 392)			Educators’ perspectives (Total number = 152)		
	Very Important N (%)	Somewhat Important N (%)	Not Important at All N (%)	Very Important N (%)	Somewhat Important N (%)	Not Important at All N (%)
Patient communication and counselling skills	210 (53.6%)	159 (40.6%)	23 (5.9%)	67 (44.1%)	85 (55.9%)	0 (0%)
Problem-solving skills	277 (70.7%)	110 (28%)	5 (1.3%)	120 (78.9%)	32 (21.1%)	0 (0%)
Dispensing procedures	208 (53.1%)	181 (46.2%)	3 (0.8%)	49 (32.2%)	103 (67.8%)	0 (0%)
Interprofessional communication skills	215 (54.8%)	175 (44.6%)	2 (0.5%)	50 (32.9%)	93 (61.2%)	9 (5.9%)
Hospital pharmacy practice	219 (55.9%)	173 (44.1%)	0 (0%)	67 (44.1%)	85 (55.9%)	0 (0%)
Community pharmacy practice	177 (45.2%)	206 (52.6%)	9 (2.3%)	120 (78.9%)	32 (21.1%)	0 (0%)
Community clinic practice (i.e., working in general practice/family doctor practice)	115 (29.3%)	240 (61.2%)	37 (9.4%)	44 (28.9%)	70 (46.1%)	36 (23.7%)

When educators were asked about their knowledge of CBS in pharmacy practice education, (28.9%, n = 44) educators described their knowledge as good, while (28.3%, n = 43) rated it as fair. Additionally, (21.7%, n = 33) educators considered it very good, and (5.9%, n = 9) rated it as excellent. However, (15.1%, n = 23) educators acknowledged that their knowledge about CBS was poor. The survey also explored awareness of available CBS tools for pharmacy practice training revealing that (78.9%, n = 120) educators were aware of such tools, whereas (21.1%, n = 32) educators were not. Interestingly, despite this awareness, only (65.1%, n = 99) educators who had actually used CBS in their teaching.

There was overwhelming support from educators for measures to reduce workload. All educators (100%, n = 152) strongly agreed/agreed with the statements *“I would like to access ready-designed scenarios”,* and *“I would like the tool to help in decreasing my workload”.*

When asked if they received enough financial support from their institutions for adopting new teaching approaches, the responses were more mixed–in response to *“My school provides adequate financial support for adopting new approaches in teaching,”* (56.6%, n = 86) educators strongly agreed/agreed, but (26.3%, n = 40) educators strongly disagreed/disagreed. Responses on technical support provision were also divided. For the statement, *“My school provides sufficient technical support when needed,”*, (53.3%, n = 81) educators strongly agreed/agreed, while (17.8%, n = 27) educators strongly disagreed/disagreed.

### 3.2 General perceptions of participants with CBS experience

Ninety-nine (65.1%) educators and 245 (62.5%) students reported that they have used CBS. A small majority [(53.5%, n = 53) educators and (63.7%, n = 157) students) reported using it often, while (44.4%, n = 44) educators and (27.8%, n = 68) students used it sometimes. Outside of scheduled school time, the frequency of use decreases significantly. Only (4%, n = 4) educators and (9.4%, n = 23) students reported using CBS often, while the majority, (79.8%, n = 79) educators and (53%, n = 130) students used it sometimes. Notably, (16.2%, n = 16) educators and (37.6%, n = 92) students reported never using CBS outside scheduled class time. Moreover, when asked for their opinion about whether CBS is used often enough in their courses, (53.5%, n = 53) educators and (63.7%, n = 156) students felt it was not used enough. In contrast, (44.4%, n = 44) educators and (27.8%, n = 68) students believed it was used the right amount, and only (2%, n = 2) educators thought it was used too often (See Table 3).

**TABLE 3:** Usage and perception among educators and students with previous experience using CBS.

Metric	Educators	Students
Survey Participants	152 (100%)	392 (100%)
Have you used CBS as part of your pharmacy practice or clinical pharmacy practice learning/teaching?		
- Yes	99 (65.1%)	245 (62.5%)
- No	53 (34.9%)	147 (37.5%)
Follow-up question for those answering “Yes”:		
“How often do you use CBS to teach/study in scheduled school time?”		
- Never	2 (2%)	21 (8.6%)
- Sometimes	44 (44.4%)	68 (27.8%)
- Often	53 (53.5%)	156 (63.7%)
“How often do you use CBS to teach/study outside scheduled school time?”		
- Never	16 (16.2%)	92 (37.6%)
- Sometimes	79 (79.8%)	130 (53%)
- Often	4 (4%)	23 (9.4%)
“Do you think CBS is used often enough in your pharmacy practice/clinical pharmacy course/unit/module?”		
- Not enough	53 (53.5%)	156 (63.7%)
- The right amount	44 (44.4%)	68 (27.8%)
- Too often	2 (2%)	0 (0%)
- Unable to judge	0 (0%)	21 (8.6%)

When educators were asked, *“How often do you encourage your students to use a computer-based simulation tool?”,* (53.5%, n = 53) educators reported that they often encouraged their students to use CBS tools, and (46.5%, n = 46) educators stated that they sometimes encouraged the use of CBS. No educators responded with “Never”.

### 3.3 Specific perspectives of participants with CBS experience

Educators and students identified similar CBS tools. Interestingly, dispensing and patient management software systems were identified alongside educational CBS (see Table 4). Educational CBS tools such as MyDispense, Pharmacy Simulator, SimPharm, SimConverse, EHR Go, and others are primarily designed to enhance academic learning, focusing on patient communication, problem-solving, and clinical skills. However, software systems like PioneerRx, OutcomesMTM, Oracle, and Fred Dispense, that are tailored for practical, industry-specific tasks, such as dispensing, inventory management, and patient outcomes tracking, were also reported by participants.

**TABLE 4:** Participants (Students and Educators) own experience using CBS in their teachings/learnings.

Metric	Educators	Students
Surveys Participants who indicated that they have previous experience utilising CBS in their teaching/learning	n = 99 (100%)	n = 245 (100%)
Name of the CBS tool most used	Educational CBS: MyDispense, SimPharm, PharmaCase, SimConverse, Virtual Patient software, Pharmacy Simulator, EHR Go, Virtual Community Placement (VCP) Professional Software Systems: PioneerRx, OutcomesMTM, Oracle, Fred Dispense	Educational CBS: MyDispense, SimPharm, PharmaCase, SimConverse, Virtual Patient software, Pharmacy Simulator, EHR Go Professional Software Systems: PioneerRx, Oracle, Fred Dispense
Last use of the CBS tool	2022-2023	2022-2023
Agreement with statements		
It has major technical issues that prevent you from completing the exercise		
- Agree/Strongly Agree	4 (4%)	21 (8.6%)
- Neither Agree/Disagree	16 (16.2%)	68 (27.8%)
- Disagree/Strongly Disagree	79 (79.8%)	156 (63.7%)
It has minor technical issues that do not prevent you from completing the exercise		
- Agree/Strongly Agree	53 (53.5%)	130 (53%)
- Neither Agree/Disagree	46 (46.5%)	92 (37.6%)
- Disagree/Strongly Disagree	0 (0%)	23 (9.4%)
Sufficient tutorials and online support on the simulator were provided		
- Agree/Strongly Agree	53 (53.5%)	111 (45.3%)
- Neither Agree/Disagree	24 (24.2%)	42 (17.2%)
- Disagree/Strongly Disagree	22 (22.2%)	92 (37.6%)
It was easy to use		
- Agree/Strongly Agree	60 (60.6%)	173 (70.6%)
- Neither Agree/Disagree	4 (4%)	43 (17.6%)
- Disagree/Strongly Disagree	35 (35.4%)	29 (11.8%)
It adequately replicates a real-world pharmacy practice experience		
- Agree/Strongly Agree	53 (53.5%)	156 (63.7%)
- Neither Agree/Disagree	22 (22.2%)	68 (27.8%)
- Disagree/Strongly Disagree	24 (24.2%)	21 (8.6%)
It is a time-efficient way to teach/study		
- Agree/Strongly Agree	46 (46.5%)	130 (53%)
- Neither Agree/Disagree	15 (15.2%)	92 (37.6%)
- Disagree/Strongly Disagree	38 (38.4%)	23 (9.4%)
It is affordable for my school/college/faculty		
- Agree/Strongly Agree	53 (53.5%)	N/A
- Neither Agree/Disagree	22 (22.2%)	N/A
- Disagree/Strongly Disagree	24 (24.2%)	N/A
It is a cost-efficient training method		
- Agree/Strongly Agree	75 (75.8%)	N/A
- Neither Agree/Disagree	8 (8.1%)	N/A
- Disagree/Strongly Disagree	16 (16.2%)	N/A
It helps in controlling the training quality		
- Agree/Strongly Agree	79 (79.8%)	N/A
- Neither Agree/Disagree	16 (16.2%)	N/A
- Disagree/Strongly Disagree	4 (4%)	N/A
It is an enjoyable way to study		
- Agree/Strongly Agree	N/A	177 (72.4%)
- Neither Agree/Disagree	N/A	39 (15.9%)
- Disagree/Strongly Disagree	N/A	29 (11.8%)
It is an engaging way to study		
- Agree/Strongly Agree	N/A	190 (77.6%)
- Neither Agree/Disagree	N/A	34 (13.9%)
- Disagree/Strongly Disagree	N/A	21 (8.6%)
It should be used more in pharmacy practice education		
- Agree/Strongly Agree	46 (46.5%)	173 (70.6%)
- Neither Agree/Disagree	15 (15.2%)	43 (17.6%)
- Disagree/Strongly Disagree	38 (38.4%)	29 (11.8%)

The survey indicated that major technical issues were not a significant barrier, with only few educators (4%, n = 4) and students (8.6%, n = 21 students) agreeing that such issues prevented them from completing exercises. However, minor technical issues were more commonly acknowledged, among educators (53.5%, n = 53) and students (53%, n = 130).

Opinions on support and ease of CBS use were mixed. While (53.5%, n = 53) educators and (45.3%, n = 111) students agreed that sufficient tutorials and online support were provided, (37.6%, n = 92) students disagreed. In terms of ease of use, (60.6%, n = 60) educators and (70.6%, n = 173) students found CBS tools easy to use, although (35.4%, n = 35) educators expressed dissatisfaction.

CBS tools were generally viewed as effective in replicating real-world pharmacy practice training, with (53.5%, n = 53) educators and (63.7%, n = 156) students agreeing on their effectiveness. CBS was also seen as a time-efficient method by (46.5%, n = 46) educators and (53%, n = 130) students, although (38.4%, n = 38) educators disagreed. Financially, CBS was perceived positively by educators, with (53.5%, n = 53) agreeing that it is affordable and (75.8%, n = 75) considering it cost-efficient. Furthermore, (79.8%, n = 79) educators felt that CBS helps control training quality.

Students were particularly positive about the engagement factor, with (72.4%, n = 177) finding CBS enjoyable and (77.6%, n = 190) agreeing it was engaging. A strong majority of students (70.6%, n = 173) and nearly half of educators (46.5%, n = 46) supported increased use of CBS in pharmacy education (see Table 4).

### 3.4 Participants’ views on the expected support level for CBS integration from different stakeholder groups

When comparing the perceptions of students and educators regarding support for CBS implementation, distinct differences emerged in how each group views the expected support levels from various stakeholders. A chi-square test confirmed that these differences are statistically significant (X^2^ (2, N = 544) = 120.96, *p* < 0.001), as detailed in Table 5.

**TABLE 5:** Participants views on the expected support level for CBS integration from different stakeholder groups.

Survey	Targeted cohort	Survey question	Not Likely N (%)	Somewhat Likely N (%)	Very Likely N (%)
Students’ Perspective Participants (N= 392 (%))	Peer Students’ Support	Do you think pharmacy students would support the implementation of computer-based simulation in your pharmacy program?	13 (4.8%)	226 (57.7%)	153 (37.5%)
	Educators’ Support	Do you think educators would support the implementation of computer-based simulation in your pharmacy program?	115 (29.3%)	246 (62.8%)	31 (7.9%)
	Institutional Support	Do you think leaders (i.e. Head of schools, deans etc) would support the implementation of computer-based simulation in your pharmacy program?	102 (26%)	261 (66.6%)	29 (7.4%)
Educators’ Perspective Participants (N= 152 (%))	Students’ Support	Do you think pharmacy students would support the implementation of computer-based simulation in your pharmacy program?	4 (2.6%)	31 (20.4%)	117 (77%)
	Peer Educators’ Support	Do you think educators would support the implementation of computer-based simulation in your pharmacy program?	13 (8.6%)	103 (67.8%)	36 (23.7%)
	Institutional Support	Do you think leaders (i.e. Head of schools, deans etc) would support the implementation of computer-based simulation in your pharmacy program?	10 (6.6%)	103 (67.8%)	39 (25.7%)

Chi-square was applied. *p* <0.05 is considered statistically significant.

Among students, (37.5%, n = 153) believed their peers were very likely to support CBS, and (57.7%, n = 226) considered it somewhat likely. This view is mirrored by educators, where (77%, n = 117) believed students were very likely to support CBS, and (20.4%, n = 31) thought it was somewhat likely, with only (2.6%, n = 4) doubting student support. However, perceptions differed when it came to educator support. Only (7.9%, n = 31) of students felt educators were very likely to support CBS, and (29.3%, n = 115) considered it unlikely. In contrast, educators were more optimistic about their peers, with (23.7%, n = 36) believing strong support was very likely and (67.8%, n = 103) finding it somewhat likely.

Regarding institutional support from leaders such as heads of schools and deans, students were less optimistic, with only (7.4%, n = 29) students believing it was very likely and (26%, n = 102) students considering it unlikely. Educators, on the other hand, were more optimistic, with (25.7%, n = 39) educators seeing strong institutional support as very likely and (67.8%, n = 103) educators finding it somewhat likely, while only (6.6%, n = 10) educators thought it unlikely.

### 3.5 Regional differences in collected responses

We compared both student and educator responses across the WHO regions, as shown in Table 6.

**TABLE 6:** Combined (Educators and Students surveys) regional analysis of CBS utilisation and implementation perceptions.

Metric / Region	Region of the Americas (AMRO)	South-East Asia Region (SEARO)	European Region (EURO)	Eastern Mediterranean Region (EMRO)	African Region (AFRO)	Western Pacific Region (WPRO)
Educators Survey Participants (n= 152, 100%)	51 (33.6%)	15 (9.9%)	25 (16.4%)	30 (19.7%)	9 (5.9%)	22 (14.5%)
Have you used CBS as part of your pharmacy practice or clinical pharmacy practice teaching? (n = 152, 100%)						
Yes - (Total number = 99 (65.1%))	36 (70.6%)	7 (42.9%)	17 (68%)	19 (63.3%)	4 (44.4%)	16 (72.7%)
No - (Total number = 53 (34.9%))	15 (29.4%)	8 (57.1%)	8 (32%)	11 (36.7%)	5 (55.6%)	6 (27.3%)
*“When considering your personal preference for using computer-based simulation. Indicate your level of agreement with each of the following statements”*						
I would be comfortable using computer-based technology in teaching (n = 152, 100%)						
Agree/ Strongly Agree - 127 (83.6%)	46 (90.2%)	5 (33.3%)	20 (80%)	25 (83.3%)	9 (100%)	22 (100%)
Neither Agree/Disagree - 14 (9.2%)	4 (7.8%)	5 (33.3%)	5 (20%)	0 (0%)	0 (0%)	0 (0%)
Disagree/Strongly Disagree – 11 (7.2%)	1 (2%)	5 (33.3%)	0 (0%)	5 (16.7%)	0 (0%)	0 (0%)
I would like to use a computer-based simulation tool to assess my students’ knowledge (n = 152, 100%)						
Agree/ Strongly Agree - 86 (56.6%)	30 (58.8%)	10 (66.7%)	15 (60%)	15 (50%)	6 (66.7%)	10 (45.5%)
Neither Agree/Disagree - 47 (30.9%)	9 (17.6%)	5 (33.3%)	8 (32%)	11 (36.7%)	3 (55.6%)	11 (50%)
Disagree/Strongly Disagree - 19 (12.5%)	12 (23.5%)	0 (0%)	2 (8%)	4 (16.7%)	0 (0%)	1 (4.5%)
*“When considering your school infrastructure to support the use of computer-based simulation”*						
My school provides sufficient technical support when needed (n = 152, 100%)						
Agree/ Strongly Agree - 81 (53.3%)	30 (58.8%)	5 (33.3%)	15 (60%)	15 (50%)	5 (55.6%)	11 (50%)
Neither Agree/Disagree - 44 (28.9%)	21 (41.2%)	3 (20%)	5 (20%)	5 (16.7%)	0 (0%)	10 (45.5%)
Disagree/Strongly Disagree - 27 (17.8%)	0 (0%)	7 (46.7%)	5 (20%)	10 (33.3%)	4 (44.4%)	1 (4.5%)
My school provides adequate financial support for adopting new approaches in teaching (n = 152, 100%)						
Agree/ Strongly Agree - 86 (56.6%)	30 (58.8%)	10 (66.7%)	15 (60%)	15 (50%)	6 (66.7%)	10 (45.5%)
Neither Agree/Disagree - 26 (17.1%)	15 (29.4%)	0 (0%)	5 (20%)	5 (16.7%)	0 (0%)	1 (4.5%)
Disagree/Strongly Disagree - 40 (26.3%)	6 (11.8%)	5 (33.3%)	5 (20%)	10 (33.3%)	3 (33.3%)	11 (50%)
Students Survey Participants (n= 392, 100%)	74 (18.9%)	30 (7.7%)	90 (23%)	100 (25.5%)	21 (5.4%)	77 (19.6%)
Have you used CBS as part of your pharmacy practice or clinical pharmacy practice learning? (n = 392, 100%)						
Yes - (Total number = 245 (62.5%))	47 (63.5%)	9 (30%)	56 (62.2%)	71 (71%)	8 (38.1%)	54 (70.1%)
No - (Total number = 147 (37.5%))	27 (36.5%)	21 (70%)	34 (37.8%)	29 (29%)	13 (61.9%)	23 (29.9%)
*“When considering your personal preference for using computer-based simulation. Indicate your level of agreement with each of the following statements”*						
I would like to use a computer-based simulation tool to assess my knowledge (n = 392, 100%)						
Agree/ Strongly Agree - 282 (71.9%)	54 (72.9%)	9 (30%)	66 (73.3%)	81 (81%)	8 (38.1%)	64 (83.1%)
Neither Agree/Disagree - 93 (23.7%)	5 (6.8%)	21 (70%)	24 (26.7%)	19 (19%)	13 (61.9%)	11 (14.3%)
Disagree/Strongly Disagree - 17 (4.3%)	15 (20.3%)	0 (0%)	0 (0%)	0 (0%)	0 (0%)	2 (2.6%)
I am comfortable with the use/integration of computer-based technology in learning (n = 392, 100%)						
Agree/ Strongly Agree - 330 (84.2%)	64 (86.5%)	27 (90%)	69 (76.7%)	88 (88%)	20 (95.2%)	62 (80.5%)
Neither Agree/Disagree - 52 (13.2%)	5 (6.8%)	3 (10%)	21 (23.3%)	12 (12%)	1 (4.8)	10 (13%)
Disagree/Strongly Disagree - 10 (2.6%)	5 (6.8%)	0 (0%)	0 (0%)	0 (0%)	0 (0%)	5 (6.5%)

Educators only in EMRO (Eastern Mediterranean) (63.3%, n = 19) reported lower usage of CBS compared to students (71%, n = 71). Conversely, educators in AMRO (Americas) (70.6%, n = 36), WPRO (72.7%, n = 16), and EURO (68%, n = 17) reported higher usage of CBS compared to students, with AMRO students at (63.5%, n = 47), WPRO students at (70.1%, n = 54), and EURO students at (62.2%, n = 56). Similarly, both SEARO (Southeast Asia) and AFRO (Africa), educators reported higher CBS usage than students, however, the reported usage was quite low overall, with SEARO educators at (42.9%, n = 7) compared to students at (30%, n = 9), and AFRO educators at (44.4%, n = 4) compared to students (38.1%, n = 8).

Educators across regions generally reported higher comfort levels with CBS compared to students. Interestingly, all educators in WPRO (n = 22) and AFRO (n = 9) expressed 100% comfort. Notably, AFRO students also reported high confidence (95.2%, n = 20), despite the overall lower CBS usage rates in the region.

Both SEARO and AFRO regions expressed concerns regarding access to technical support, with (46.7%, n = 7) of SEARO and (44.4%, n = 4) of AFRO educators disagreeing with the statement, “My school provides sufficient technical support when needed.” EMRO followed closely with (33.3%, n = 10) of educators expressing similar concerns. Additionally, both SEARO and AFRO raised issues regarding financial support, with 33.3% of respondents in SEARO (n = 5) and AFRO (n = 3) disagreeing. EMRO again matched this concern with 33.3% (n = 10). Surprisingly, WPRO, which includes high-income countries such as (Australia), had the highest percentage of disagreement at 50% (n = 11).

## 4 Discussion

This study explored the perceptions and experiences of pharmacy educators and students across various global regions regarding the integration and utilisation of CBS in pharmacy practice education. The findings provide significant insights into the current state of CBS adoption, and the barriers impacting its widespread implementation.

The findings indicate a significant gap between the recognised value of CBS and its practical application within pharmacy curricula, with 81 educators (53.5%) and 156 students (63.7%) reporting insufficient usage. Similar to findings in broader healthcare education, educational technologies are often underutilised despite their proven benefits (Unsworth, 2020). These results suggest initiatives to improve adoption of these technologies may be well received by students and educators alike, especially if the solution is capable of training students in their prioritised skills (clinical problem-solving and communication skills) and can model both community and hospital practice.

Educators also indicated overwhelmingly that workload factors were one of their main priorities. They indicated a desire for ready-made scenarios, and solutions which reduce workloads. This is likely a significant issue for most of the existing CBS solutions which require educators to undertake works to both author new case-based scenarios and integrate these scenarios into their curriculum. Based on this feedback solutions which include features to minimise or reduce overall workload, such as off the shelf scenarios and curriculum content, automated assessments/grading, and those that leverage generative artificial intelligence, are likely to be well received by educators.

The barriers to CBS implementation are multifaceted, encompassing technical, financial, and institutional challenges. While major technical issues were not reported as significant barriers, the presence of minor technical problems that were encountered by 130 students (53%) and 53 educators (53.5%), suggests ongoing challenges that can disrupt the learning experience.

Financial constraints and insufficient technical support (reported by 86 educators (56.6%) and 81 educators (53.3%), respectively), requires targeted resource allocation. While the results indicate that a majority of educators who had experience with using CBS, view CBS as both affordable (53.5%, n = 53) and cost-efficient (75.8%, n = 75), there are still underlying concerns among some educators regarding the initial costs and time investment required for its integration into the curriculum. These concerns may stem from the broader context of budget constraints and inadequate infrastructure, challenges that are well-documented in the literature when integrating new educational tools into academic settings (Haleem et al., 2022; Graham, 2013) and, where the upfront costs of new technology can deter its adoption (O’Doherty et al., 2018).

Research to demonstrate the long-term benefits and cost-effectiveness of CBS could help alleviate the above concerns and foster broader acceptance among educators. Additionally, investing in robust IT support systems and adequately funding computer resources to mitigate minor technical issues is likely to improve uptake and student acceptance within institutions.

Both educators and students reported high levels of comfort with using CBS, which aligns with existing studies indicating a general positive attitude toward technology-enhanced learning in healthcare education (Bennett and Maton, 2010). However, it is noteworthy that a small but significant proportion of both groups expressed neutrality or disagreement regarding their comfort with CBS. This variation may be due to differing levels of digital literacy, as suggested by previous research highlighting the impact of digital competence on the adoption of educational technologies (Bennett and Maton, 2010). Tailored training and support to address these differences, and CBS features which help to support and onboard new users, could enhance the effectiveness and acceptance of CBS across the board.

There was a slight divide in perceived likelihood of CBS integration initiatives being supported–students generally believed educators and institutions were less likely to lend their support, while educators were a little more optimistic, with a greater percentage of “Very Likely” responses and fewer “Not Likely” responses. This student scepticism may stem from past experiences where new initiatives were introduced without sufficient follow-through, a common issue in educational reform efforts (Rogers et al., 2019). This gap also may reflect a communication barrier between students and faculty or a lack of transparency in institutional decision-making processes (Chen et al., 2020). On balance however, both students and educators tended to agree that such initiatives were at least “Somewhat Likely” to be supported across the board, with no clear trend to suggest that any particular stakeholder group would be in firm opposition.

Although this study was not designed to capture this data, we do note that a number of educators indicated they make use of professional software systems like PioneerRx, FredDispense, and Oracle as CBS substitutes. While offering practical benefits by helping to produce practice ready graduates, we would note some minor concerns with this approach. These tools, designed for specific industry tasks (e.g., dispensing, stock management, record keeping), risk narrowing students’ learning by focusing on procedural skills, rather than fostering a patient focus. Unlike tools designed for educational purposes, such as MyDispense, Pharmacy Simulator and SimPharm, dispensing software are also not designed to provide user-friendly assessment and feedback suitable for students. Proper use of these tools should be scaffolded with sufficient instructional guidance and other exercises, such as patient counselling role-plays, to be utilised effectively. This approach presents its own set of logistical and resourcing challenges. We would suggest that educational institutions critically assess this balance to ensure that these tools complement rather than dominate the curriculum, preserving the core aim of developing well-rounded, adaptable pharmacists.

It is unsurprising that in developing regions such as Southeast Asia (SEARO) and Africa (AFRO), access to technical and financial support presents a greater concern. These findings align with broader trends in the literature that highlight the ongoing challenges faced by educational institutions in low- and middle-income regions, where limited infrastructure and resources often impede the integration of advanced educational technologies like CBS (Naresh and Reddy, 2015). The concern raised by respondents in the Western Pacific region (WPRO) likely reflects the constrained higher education landscape in Australia, where many universities have been grappling with financial difficulties due to reduced international student numbers following the COVID-19 pandemic (Almaiah et al., 2020; Carnegie et al., 2022). This financial strain has led to widespread budget cuts, staffing reductions, and the scaling back of technological investments in teaching and learning (Carnegie et al., 2022). Such factors may have contributed to the concerns around financial and technical support for CBS integration in this region.

## 5 Strengths, limitations and future research

This study builds on previous research by providing a more comprehensive, global perspective on CBS adoption in pharmacy education. Unlike earlier studies that primarily focused on specific tool integration and specific institutions, this research captured insights from 152 educators from 38 countries, and 392 students from 46 countries, spanning six WHO regions (AFRO, AMRO, EMRO, EURO, SEARO, and WPRO). Moreover, the widespread support for CBS from both educators and students suggests that greater inclusion of CBS in daily pharmacy education practice could enhance student skills in areas such as communication and problem-solving, while also addressing educator concerns about workload.

The reliance on self-reported data from surveys may introduce bias, since the topic may have primarily attracted individuals who are already interested in CBS or supportive of its use. This self-selection bias could potentially impact the results towards more positive perceptions of CBS, limiting the generalisability of the findings.

We also acknowledge that participants’ knowledge and experience with CBS does vary. Some participants may have only a passing interest in the technology, while others may have extensive experience using it. This variability in familiarity could impact the depth of their perspectives, with those who have limited experience potentially offering narrower or unsubstantiated views. Many participants were also found to have only used a single CBS tool, which restricts their ability to provide a comprehensive assessment of CBS technology more generally.

A key limitation of this study is the regional imbalance, especially the low participation from the African Region (AFRO), with only 5.9% of educators and 5.4% of students represented. This limits the reliability and generalisability of the data for those areas. While this study analysed data at the regional level, significant country-level differences within regions may exist, particularly in regions like Europe or the Americas where economic and educational development varies considerably. It is worth noting that certain countries dominated regional responses. For instance, in the educators’ survey, the USA contributed 82.4% of AMRO responses, India 60% of SEARO, Egypt 26.7% of EMRO, Ireland 32% of EURO, and Australia 50% of WPRO. This made it difficult to compare between countries in the same region, as sample sizes were diminished at this level of granularity. Future research should aim for more balanced regional representation through targeted recruitment in underrepresented regions to offer more granular insights.

We also attempted to complement the quantitative data with qualitative insights by asking participants to voluntarily share additional comments. However, the response rate for these qualitative inputs was very low, limiting our ability to explore more in-depth perspectives (Andrade, 2020). The scarcity of qualitative feedback highlights the challenges of relying on voluntary responses in survey-based research, particularly with global recruitment. Future research could benefit from employing dedicated qualitative methods, such as interviews or focus groups with educators and leaders, to gain further insights to complement the results of our surveys. This research could potentially explore socio-cultural factors affecting CBS adoption across different educational settings. Understanding these influences may be crucial for developing effective, inclusive strategies tailored to the diverse needs of global educational environments and help guide the future development of CBS systems.

## 6 Conclusion

This study provides valuable insights into the global perceptions of CBS implementation in pharmacy education, highlighting its potential and the barriers to broader adoption. Both educators and students expressed comfort with CBS and recognised its value in developing essential skills, such as communication and problem-solving. However, significant gaps in its utilisation were identified, with educators particularly highlighting workload concerns and a desire for CBS to help reduce this. Financial constraints and limited technical support were also notable barriers, especially in SEARO, AFRO, and WPRO.

The regional disparities in CBS adoption suggest that, while there is strong support for integrating CBS into pharmacy curricula, targeted efforts are needed to address the unique challenges in different regions. Based on our study’s findings, educators, policymakers and institutions seeking to implement CBS should ensure they have adequate resources, including appropriate computer hardware, technical support, and training, to increase the chances of success. CBS developers should focus on creating tools that support students in practising problem-solving and communication skills in both community and hospital settings, and prioritise features which might reduce educators’ workloads.

By addressing these barriers, pharmacy education can fully harness the potential of CBS to enhance empirical learning and better prepare students for real-world clinical practice.

## Data Availability

The raw data supporting the conclusions of this article will be made available by the authors, without undue reservation.
