# Supplementary material for: Exploring global perspectives on the use of computer-based simulation in pharmacy education: a survey of students and educators
Source: Front Pharmacol. 2024 Oct 15;15:1494569. doi: 10.3389/fphar.2024.1494569 (PMC11518731; doi:10.3389/fphar.2024.1494569)
Supplement: Supplementary file 1 [file DataSheet1.PDF]

Appendix A – Students' Survey Questionnaire

Instructions: This survey may take up to 15 minutes to complete.

<u>Section 1</u>

<u>A. Choose one of the following answers</u>

1. How long is your pharmacy program at the University (full-time equivalent years)?
(Drop-down list with numbers 1-6)
2. How many years are left for you to finish/complete your university pharmacy program?
(Drop-down list with numbers 1-6)
3. Have you used computer-based simulation as part of your pharmacy practice or clinical pharmacy practice education? (Yes, No)
**If answered Yes – section B is required to be answered.*

<u>B. Please select your answer from the drop-down lists</u>

1. How often do you use a computer-based simulation tool to study outside scheduled school time?
(Drop down list of NEVER, SOMETIMES, OFTEN)
2. How often do you use a computer-based simulation tool in scheduled classes at your school?
(Drop down list of NEVER, SOMETIMES, OFTEN)
3. Do you think computer-based simulation is used often enough in your pharmacy practice/clinical pharmacy course/module?
(Drop down list of Not enough, the right amount, too often, Unable to judge)

<u>Section 2</u>

Using the categories below, choose the most appropriate response for each item:

1. When considering the computer-based simulation: Indicate how you rate the importance of each of the following elements *(very important=5 to not important at all=1).*

1. Realistic graphics
2. Ease of use
3. Bug-free experience
4. Availability of tech-support
5. Detailed feedback
6. Engaging content
7. Fun/enjoyable content

2.2 When considering the training focus for a computer-based simulation: Indicate how you rate the importance of each of the following elements (strongly agree=5 to strongly disagree=1).

1. Patient communication and counselling skills
2. Problem-solving skills
3. Dispensing procedure
4. Interprofessional communication skills
5. Hospital pharmacy practice
6. Community pharmacy practice
7. Community clinic practice (i.e. working in general practice/family doctor practice)

2.3 When considering your personal preference for using computer-based simulation. Indicate your level of agreement with each of the following statements (strongly agree=5 to strongly disagree=1).

1. I would like to use a computer-based simulation tool to assess my knowledge
2. I would like to use a computer-based simulation tool in the classroom
3. I would like to use a computer-based simulation tool to supplement my classroom study

2.4 When considering your school infrastructure to support the use of computer-based simulation. Indicate your level of agreement with each of the following statements. (strongly agree=5 to strongly disagree=1)

1. My school provides adequate access to computers
2. My school provides a reliable internet connection
3. My school embraces innovation in teaching

2.5 When considering your ability to use a computer-based simulation to study at home. Indicate your level of agreement with each of the following statements. (5=strongly agree to 1=disagree)

1. I have access to a suitable personal device (i.e., PC, laptop, tablet, phone) at home
2. I have a reliable personal internet connection at home
3. I am comfortable using computer-based technology in my study

Section 3

Help: There will be questions regarding your perceptions and experience using one or more computer-based simulation tools.

3.1 How many computer-based simulation platforms have you used when studying Pharmacy practice/Clinical pharmacy?
3.2 What is the name of the tool you have used?
3.3 When did you last use this tool?
3.4 Based on your previous experience with the above-named computer-based simulation platform, indicate your level of agreement with each of the following statements (strongly agree=5 to strongly disagree=1).
Help: "Technical issues": an unexpected problem with the software such as (lag, bugs and glitches) that result in interfering with the program's performance.

1. It has major technical issues that prevent you from completing the exercise
2. It has minor technical issues that do not prevent you from completing the exercise
3. Sufficient tutorials and online support on the simulator were provided
4. It was easy to use
5. It adequately replicates a real-world pharmacy practice experience
6. It is an enjoyable way to study
7. It is a time-efficient way to study
8. It is an engaging way to study
9. It should be used more in pharmacy practice education

3.5 If this simulation platform was missing an important feature, please, explain it below.
3.6 What did you like most about this platform?
3.7 What did you most dislike about this platform?

Section 4

4.1. Do you think pharmacy students would support the implementation of computer-based simulation in your pharmacy program?

Very likely	Somewhat likely	Not Likely

4.2. Can you please specify why did you choose this answer regarding the Students' stakeholder group?
4.3. Do you think educators would support the implementation of computer-based simulation in your pharmacy program?
Help: Educators: i.e. Teachers, lecturers...etc

Very likely	Somewhat likely	Not Likely

4.4. Can you please specify why did you choose this answer regarding the educators' stakeholder group?

4.5. Do you think leaders would support the implementation of computer-based simulation in your pharmacy program?

Help: leaders: i.e. Heads of schools, deans...etc

Very likely	Somewhat likely	Not Likely

4.6. Can you please specify why did you choose this answer regarding the leaders' stakeholder group?

4.7. Do you think any other barriers are preventing your school from implementing computer-based simulation which has not been covered by the previous questions?

4.8. Can you please discuss those barriers?

Appendix B – Educators' Survey Questionnaire

Instructions: This survey may take up to 15 minutes to complete.

<u>Section 1</u>

<u>A. Choose one of the following answers</u>

Which is the primary language used in your course?

English or Other options
(If the participant chooses (OTHER) a drop-down list of: Different languages will appear)

<u>B. Please select your answer from the drop-down lists</u>

1. In what country do you teach?
(Drop down list of: Different countries)

2. How many years have you been teaching your course/module?
(Drop down list with average of: 0-1 yrs., 1-3 yrs., 3-6 yrs., 6-10 yrs., 10+ yrs.)

3. What is your school/college/faculty size (estimated number of students)?
(Drop down list of: 50 or less, 100 or less, 200 or less, 300 or less, more than 300).

4. How would you describe your knowledge about computer-based simulation in pharmacy practice education?
(Drop down list of Excellent, Very Good, Good, Fair, Poor).

5. Are you aware of any available computer-based simulation in pharmacy practice training? (Yes/No)

7. Have you used computer-based simulation as part of your pharmacy practice or clinical pharmacy teaching? (Yes/No)

<u>C. Please select your answer from the drop-down lists</u>

1. How often do you encourage your students to use a computer-based simulation tool?
(Drop down list of: NEVER, SOMETIMES, OFTEN)

2. How often do you use a computer-based simulation tool in scheduled classes at your school/college/faculty?
(Drop down list of: NEVER, SOMETIMES, OFTEN)

3. How often do you encourage your students to use computer-based simulation in their independent study?
(Drop down list of: NEVER, SOMETIMES, OFTEN)

4. Do you think computer-based simulation is used often enough in your course/module?

(Drop down list of: Not enough, the right amount, too often, Unable to judge)

Section 2

Using the categories below, choose the most appropriate response for each item:

1. When considering the use of computer-based simulation tools in teaching pharmacy practice/clinical pharmacy/related training, please indicate how you rate the importance of each of the following elements *(very important=5 to not important at all=1).*

1. Realistic graphics
2. Ease of use
3. Bug-free experience
4. Availability of tech-support
5. Detailed feedback
6. Engaging content
7. Fun/enjoyable content
8. Continuously updated content (i.e. when new guidelines are published)

2.2 When considering the training focus for a computer-based simulation: indicate how you rate the importance of each of the following elements. (strongly agree=5 to strongly disagree=1).

1. Patient communication and counselling skills
2. Problem-solving skills
3. Dispensing procedure
4. Interprofessional communication skills
5. Hospital pharmacy practice
6. Community pharmacy practice
7. Community clinic practice (i.e. working in general practice/family doctor practice)

2.3 When considering your personal preference in using computer-based simulation, indicate your level of agreement with each of the following statements. (strongly agree=5 to strongly disagree=1).

1. I am comfortable using computer-based technology in teaching
2. I have the freedom to incorporate technology in the curriculum the way I want
3. I find it challenging to fit activities that use computer-based simulation into pharmacy practice training

4. I would like to use a computer-based simulation tool to assess my students' knowledge
5. I would like to be able to customise the computer-based simulation tool
6. I would like to access ready-designed scenarios
7. I would like the tool to help in decreasing my workload
8. I find the computer-based simulation platform that I am using, meet my needs

2.4 When considering your school infrastructure to support the use of computer-based simulation. Indicate your level of agreement with each of the following statements. (strongly agree=5 to strongly disagree=1)

1. My school provides adequate access to computers
2. My school provides a reliable internet connection
3. My school provides sufficient technical support when needed
4. My school provides adequate financial support for adopting new approaches in teaching
5. My school embraces innovation

Section 3

Help: There will be questions regarding your perceptions and experience using one or more computer-based simulation tools.

3.1 How many computer-based simulation platforms have you used to teach pharmacy practice/clinical pharmacy/related training?
3.2 What is the name of the tool you have used?
3.3 When did you last use this tool?
3.4 Based on your previous experience with the above-named computer-based simulation platform, indicate your level of agreement with each of the following statements (strongly agree=5 to strongly disagree=1).
Help: "Technical issues": an unexpected problem with the software such as (lag, bugs and glitches) that result in interfering with the program's performance.

1. It has major technical issues that prevent you from completing the exercise
2. It has minor technical issues that do not prevent you from completing the exercise
3. Sufficient tutorials and online support on the simulator were provided
4. It was easy to use
5. It adequately replicates a real-world pharmacy practice experience
6. It is an enjoyable way to study
7. It is a time-efficient way to study

8. It is an engaging way to study
9. It should be used more in pharmacy practice education

3.5 If this simulation platform was missing an important feature, please, explain it below.
3.6 What did you like most about this platform?
3.7 What did you most dislike about this platform?

Section 4

4.1. Do you think pharmacy students would support the implementation of computer-based simulation in your pharmacy program?

Very likely	Somewhat likely	Not Likely

4.2. Can you please specify why did you choose this answer regarding the Students' stakeholder group?
4.3. Do you think educators would support the implementation of computer-based simulation in your pharmacy program?
Help: Educators: i.e. Teachers, lecturers...etc

Very likely	Somewhat likely	Not Likely

4.4. Can you please specify why did you choose this answer regarding the educators' stakeholder group?
4.5. Do you think leaders would support the implementation of computer-based simulation in your pharmacy program?
Help: leaders: i.e. Heads of schools, deans...etc

Very likely	Somewhat likely	Not Likely

4.6. Can you please specify why did you choose this answer regarding the leaders' stakeholder group?
4.7. Do you think any other barriers are preventing your school from implementing computer-based simulation which has not been covered by the previous questions?
4.8. Can you please discuss those barriers?
